# Zona Pellucida like Domain Protein 1 (ZPLD1) Polymerization Is Regulated by Two Distinguished Hydrophobic Motifs

**DOI:** 10.3390/ijms232213894

**Published:** 2022-11-11

**Authors:** Marie Isabell Knepper, Jens Dernedde

**Affiliations:** 1 Institut für Chemie und Biochemie, Freie Universität Berlin, 14195 Berlin, Germany; 2 Institut für Laboratoriumsmedizin, Klinische Chemie und Pathobiochemie, Charité-Universitätsmedizin Berlin, Corporate Member of Freie Universität Berlin and Humboldt-Universität zu Berlin, 13353 Berlin, Germany

**Keywords:** ZPLD1, ZP proteins, hydrogel, vestibular organ, protein-protein interaction, polymerization, protein processing, cupula

## Abstract

Zona Pellucida Like Domain 1 Protein (ZPLD1) is a main component of the cupula, a gelatinous structure located in the labyrinth organ of the inner ear and involved in vestibular function. The *N*-glycosylated protein is likely able to organize high-molecular-weight polymers via its zona pellucida (ZP) module, which is common for many extracellular proteins that self-assemble into matrices. In this work, we confirmed that ZPLD1 can form multimers while setting up a cellular model leveraging Madin–Darby canine kidney (MDCK) cells to study protein polymerization. We identified two motifs within ZPLD1 which regulate its polymerization and follow previously published conserved regions, identified across ZP proteins. Mutational depletion of either one of these modules led to diminished or abnormal polymer formation outside of the cells, likely due to altered processing at the plasma membrane. Further, intracellular polymer formation was observed. Proteolytic cleavage during secretion, separating the regulatory motif located distinct of the ZP module from the mature monomer, seems to be necessary to enable polymerization. While the molecular interactions of the identified motifs remain to be proven, our findings suggest that ZPLD1 is a polymer forming ZP protein following an orchestrated mechanism of protein polymerization to finally build up a gelatinous hydrogel.

## 1. Introduction

Zona Pellucida Like Domain Protein 1 (ZPLD1), also known as Cupulin, is a 45 kDa glycoprotein initially identified as a major part of the cupula, located in the labyrinth organ of the inner ear (Supplementary Figure S1) [1].

The cupula functions as a sensory hydrogel in the liquid-filled cavity at the basis of the semicircular canals. The structure is fixed at the roof of the ampulla and rides on a barrel-like structure, the crista ampullaris. There, the gelatinous cupula is connected with the underlying neuroepithelium by kino- and stereocilia growing out from the top of hair cells. The hair cells are stimulated upon torsional acceleration of the head and corresponding rotation of the inner ear fluid. The resulting deflection of the cupula and subsequent bending of the cilia opens mechanosensitive ion channels of the hair cells by enabling a potassium influx and set up of a generator potential [2,3,4]. The afferent bipolar nerve exhibits an alteration of the action potential rate and transmits the signal further towards the brain. This stimulation mechanism is impeded once the cupula is leaked or detached from its roof [4].

Malformations in the ampulla are therefore considered as a possible explanation for the sudden loss of vestibular function. Experiments in pigeons, investigating mechanical detachment of the cupula from the roof of the ampulla support this assumption [5].

To date the biological function of ZPLD1 as part of the cupula is not yet well studied. However, loss of structural integrity of the cupula might be caused by a lack of structural material production [6]. Correspondingly, spontaneous mutations in the mouse ZPLD1 gene have been shown to lead to circling behavior of mutant mice, thereby indicating balance dysfunction [7]. ZPLD1−/− mice exhibit normal hearing function and their otolithic organs appear normal while only loss of sensory input for rotary movements was observed [7].

In humans, a ZPLD1 gene mutation leading to a 2.5-fold decreased expression level has been described in a patient exhibiting cerebral cavernous malformations (CCM). Interestingly, no alteration of vestibular function was reported for the patient [8]. Studies, searching for a causal context of aberrations of diastolic blood pressure reported a region containing the ZPLD1 gene as most significant chromosomal region [9]. These observations are particularly interesting as they suggest that ZPLD1 might be part of a complex signaling pathway implicated in CCM and blood vessel formation and may exhibit other functions besides structuring the cupula.

In addition, several genome-and/or transcriptome wide analyses which postulate ZPLD1 gene mutations related to childhood obesity, pancreatic cancer, sensory nerve disturbances and breast cancer metastasis suggest possible roles e.g., for cell–cell adhesion, migration and (nervous system) development [10,11,12,13]. The multitude of possible tasks for ZPLD1 thereby mirrors the diversity of ZP protein function, from acting as structural components of the mammalian egg coat (the zona pellucida) and other polymeric structures to serving as receptor, tumor suppressor or extracellular network to capture pathogens [14,15].

ZP proteins are extracellular glycoproteins containing a zona pellucida (ZP) module, a structural element consisting of ~260 amino acids with eight conserved cysteine residues [16]. Many ZP proteins are involved in the formation of filaments and/or matrices, which is consistent with the role of the module in protein polymerization [14]. Numerous studies suggest a common role and function of the bipartite ZP module with its N-terminal and C-terminal domains (ZP-N and ZP-C) linked via an interdomain that structurally organizes the module in a distinct manner [17]. Other common features of most ZP proteins are a membrane anchoring domain, a conserved consensus cleavage site (CCS) and two hydrophobic patches [14]. These hydrophobic patches, one within the ZP module (internal hydrophobic patch/IHP) and one located distal of the ZP module (external hydrophobic patch/EHP) seem to play a particular relevant role to regulate protein assembly [18]. Cleavage of ZP precursors during secretion was proposed to lead to a loss of the regulatory EHP, thereby enabling the released mature polypeptide to assemble [18]. Structural insights based on X-ray studies and cryo-EM models of Uromodulin revealed, that ZP module function relies on the ability to support homodimerization [19,20,21]. Homodimers, resulting from an interplay between IHP and EHP of two separate molecules present a conformation which seems to be crucial for the described cleavage and might further prevent intracellular polymerization [21]. For some ZP proteins, additional structural elements of the interdomain linker have been shown to be of high relevance and involved in maintaining this polymerization competent protein conformation [21]. The organization and type of the linker seems to play a significant role for polymeric assembly of these ZP proteins, enabling conformational changes and domain swapping [21,22]. These mechanisms facilitate the well-orchestrated scenario of IHP and EHP head-to-tail module assembly and the formation of polymers [20,22].

In this work, we aim to investigate if ZPLD1, a protein which does not display a high identity to classical ZP proteins, is able to multimerize and therefore likely accounts for polymeric assembly of the gelatinous cupula. Furthermore, we want to assess if we can identify the regulatory hydrophobic peptide regions based on their conserved structure and amino acid characteristics to understand their role for ZPLD1 maturation.

## 2. Results

### 2.1. Transformed MDCK Cells Express Physiological Active ZPLD1

**Figure 1:**
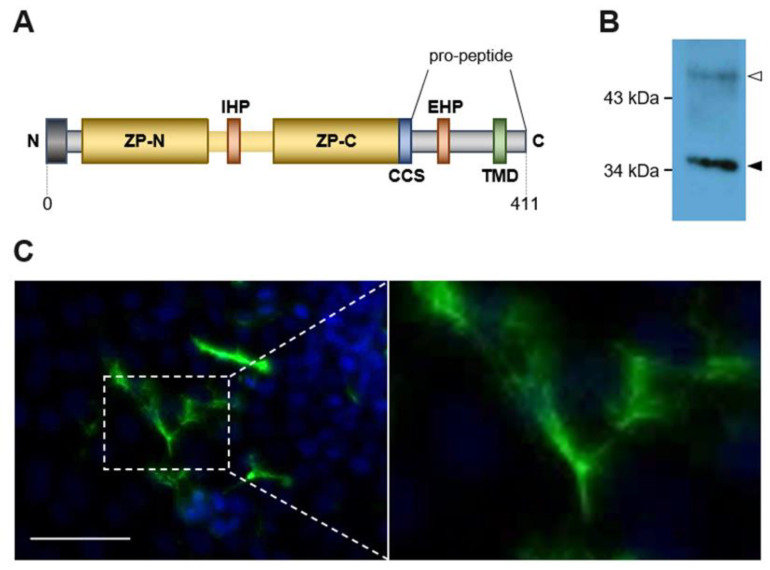
MDCK cells as a model to study ZPLD1. (**A**) Scheme of zebrafish ZPLD1 structure expected to contain a N-terminal signal peptide, a bipartite Zona Pellucida (ZP) module (including ZP-N and ZP-C domains) and a C-terminal pro-peptide [1]. The internal (IHP) and external (EHP) hydrophobic patches, the consensus cleavage site (CCS) and the transmembrane domain (TMD) are also indicated; (**B**) Western blot of PNGase *F* treated ZPLD1, purified from cell culture media of transfected MDCK cells. ZPLD1 still containing *N*-glycans (white arrowhead) shows up at higher molecular weight and a less discrete protein band compared to fully deglycosylated protein (black arrowhead); (**C**) Immunofluorescence image of non-permeabilized MDCK cells expressing Strep-tagged ZPLD1 stained with anti-Strep antibodies. Polymers formed by the protein are clearly detected on the cell surface, likely consisting of matrices or bundles of single filaments; scale bar, 50 µm.

Amino acid identity of ZPLD1 to classical ZP proteins is not very striking and only based on certain especially relevant conserved amino acids. For example, overall sequence identity between zebrafish ZPLD1 and the well-studied ZP proteins (including structural details) human Uromodulin and mouse Zona pellucida sperm-binding protein 2 is less than 15% and less than 12% respectively. At the same time however, interspecies identity between the zebrafish, salmon and human adds up to more than 70% (Supplementary Figure S2). As for other ZP proteins, we hypothesized that cleavage at a specific site at the C-terminus of ZPLD1 releases the interaction between two hydrophobic regions, thereby leading to a conformational activation of the ZP module and subsequent protein polymerization. One of these hydrophobic patches is proposed to be located within the ZP module and is therefore called internal hydrophobic patch (IHP) while the other is expected to be located outside of the ZP module as part of the pro-peptide and is therefore called external hydrophobic patch (EHP) (Figure 1A).

To confirm that ZPLD1 is able to form homopolymers and to annotate the position and role of its hydrophobic patches, a suitable cellular expression system is wanted. Finally, we transfected Madin–Darby Canine Kidney (MDCK) cells that have been previously shown to express, process and secrete the polymer forming ZP protein Uromodulin in an active form [23]. To facilitate ZPLD1 protein purification a Strep-tag with a short linker sequence was inserted 5′ to the conserved CCS between amino acids K_315_ and R_316_. Transformed MDCK cells secreted Strep-tagged ZPLD1. The protein was further purified by affinity chromatography from the culture supernatant and separated by gel-electrophoresis after enzymatic removal of *N*-glycans. ZPLD1 migrates at about 35 kDa similar to ZPLD1 extracted from salmon cupulae (Figure 1B) [1]. Obviously, the tag insert has no adverse effect on protein maturation. ZPLD1 assembles into extracellular polymers on the surface of transfected MDCK cells as observed by immunofluorescence analyses (Figure 1C). Visible polymeric structures appear as bundles or matrices likely composed of single filaments.

### 2.2. ZPLD1 Proteins Lacking the EHP or IHP Are Not Properly Processed

**Figure 2:**
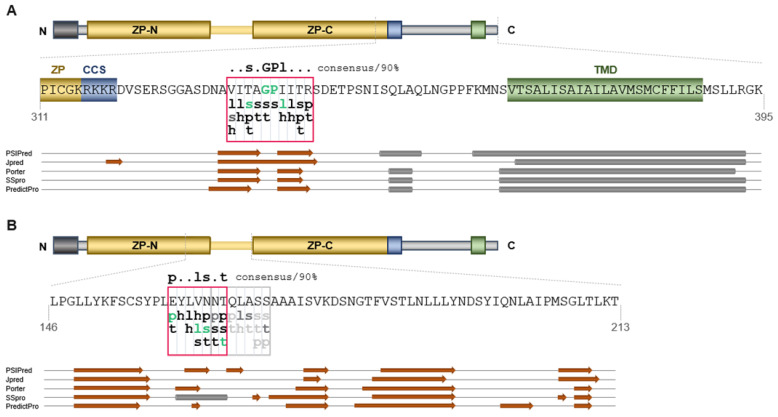
Identification of the putative IHP and EHP motifs within in ZPLD1 sequence. Scheme, detailed amino acid sequences, and annotated secondary structure predictions are shown for the EHP (**A**) and IHP (**B**). The signal peptide is shown as a dark grey box, the ZP module is shown in yellow and is likely divided in ZP-N and ZP-C domains. The consensus protease cleavage site (CCS) is shown in blue and the transmembrane domain (TMD) in green. The predicted hydrophobic patches are framed in red. Consensus with the amino acid grouping-set sequences for EHP (..s.GPl…/consensus 90%) and IHP (p..ls.t/consensus 90%) described by Schaeffer et al. is indicated as green lowercase letters [23]. The lowercase letters indicate the following grouping-sets: l, aliphatic (I, L, V); s, small (A, C, D, G, N, P, S, T, V); h, hydrophobic (F, H, I, L, M, V, W, Y); p, polar (C, D, E, H, K, N, Q, R, S, T); and t, turn-like (A, C, D, E, G, H, K, N, Q, R, S, T). Secondary structure predictions were performed with five different programs (for details see Section 4). β-strands are depicted as orange arrows, α-helices as gray cylinders.

The EHP and IHP motifs are reported to be conserved in different ZP proteins regarding their secondary structure but also, to a lesser extent, with respect to the consecutive composition of hydrophobic, aliphatic, small, turnlike and polar amino acids [23]. Based on these characteristics the amino acid sequence of ZPLD1 was analyzed. For the secondary structure prediction five different computational programs were applied (Figure 2). There is exactly one sequence area (_333_VITAGPIITR_342_) at the C-terminus of ZPLD1 that matches the corresponding consensus grouping-set sequence (..s.GPl…) for the EHP. In line with the reported high degree of secondary structure conservation in ZP proteins, the putative EHP of ZPLD1 configures two conserved β-strands within an unstructured region between the cleavage site (CCS) and the membrane anchoring domain (TMD) [23]. For the IHP two overlapping sequence areas (_260_EYLVNNT_266_ and _265_NTQLASS_271_) were identified, that match the consensus grouping-set sequence (p..ls.t). However, only the N-terminal sequence seems to form a hydrophobic β-strand as suggested feature of secondary structure conservation [23]. We therefore propose here the allocation of the IHP of ZPLD1.

Mutagenesis studies were performed to prove whether the identified sequence regions indeed correspond to the hydrophobic motifs and to better understand if their function for protein polymerization resembles the mechanism described for other ZP proteins.

**Figure 3:**
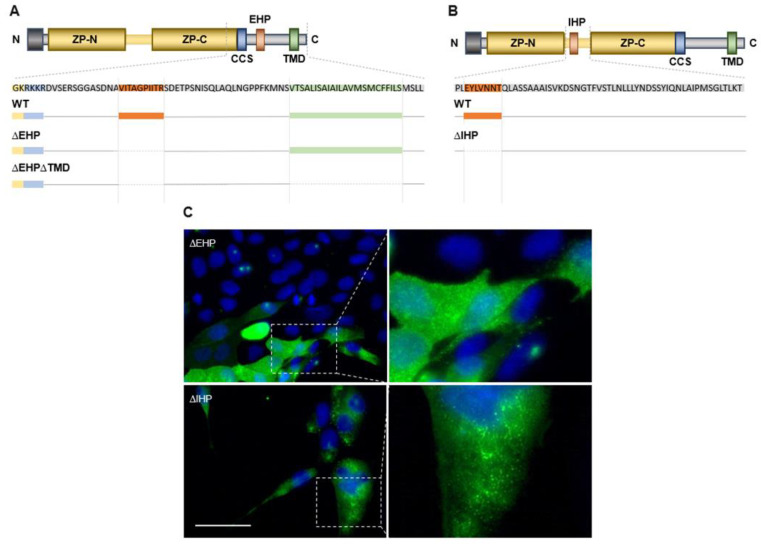
Polymerization defect in mutants lacking putative EHP and IHP sequences. (**A**,**B**) Domain structure and sequence excerpt of ZPLD1. The putative EHP and IHP motifs are highlighted in red, and they were completely deleted in the ∆EHP and ∆IHP variants. WT: wild type; (**C**) Immunofluorescence analysis showing mutant ZPLD1 on the cell surface of stably transfected MDCK cells fixed with PFA. Both variants are trafficked to the plasma membrane, but unlike wild type protein they do not polymerize properly. ∆IHP forms abnormally short and less organized polymers while ∆EHP does not form polymeric structures at all and seems to be stuck unprocessed in the plasma membrane. Scale bar, 50 µm.

Deletion constructs (Figure 3A,B) lacking the entire respective sequences were expressed in MDCK cells and characterized for their phenotype via microscopy (Figure 3C). ZPLD1 deficient of its putative EHP does not form extracellular filaments and exhibit impaired secretion. The protein seems to stick in the cytoplasmic membrane without polymerizing (Figure 3C). For the variant lacking the putative IHP motif polymer formation is impaired as well. Only abnormally short and less structured filaments might be suspected at the plasma membrane of the cells. Secretion of the variant lacking the putative IHP sequence seems not to be impaired as compared to the variant lacking the EHP. These findings support our hypotheses regarding the positioning, sequence, and function of ZPLD1′s hydrophobic patches and are in line with the processing of Uromodulin [23].

### 2.3. Depletion of EHP or IHP Leads to the Formation of Intracellular Polymeric Structures

**Figure 4:**
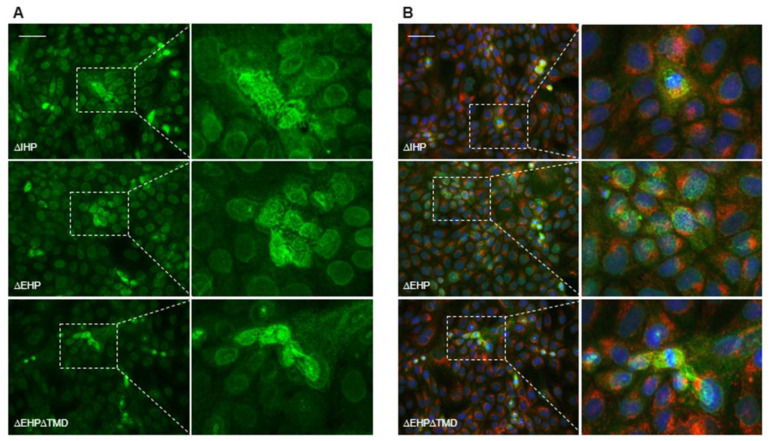
Deletion of the EHP or IHP lead to the formation of intracellular polymers. (**A**) Immunofluorescence analysis of permeabilized MDCK cells stably expressing different ZPLD1 mutant isoforms. In contrast to findings for Uromodulin [23], intracellular assembly of mutant protein can be observed for all three investigated variants. Bar, 50 µm; (**B**) Immunofluorescence of MDCK cells stably expressing ZPLD1 IHP and EHP mutant proteins. Permeabilized cells were incubated with anti-Strep, anti-GM130 Golgi marker and DAPI. The presented merged pictures show Strep (ZPLD1) and GM130 signals in green and red, respectively. DAPI cell nucleus staining is shown in blue. Intracellular filaments formed by the mutant protein do not colocalize with GM130. Bar, 50 µm.

Schaeffer et al. described intracellular polymer formation for soluble Uromodulin lacking one of its hydrophobic patches and its membrane anchor [23]. This finding supported the hypothesis that the EHP and IHP play an important role for regulation and prevention of intracellular polymerization thereby enabling proper processing and secretion. To proof this for ZPLD1 we created an EHP mutant lacking the TMD domain (Figure 3A) and observed the same phenotype with intracellular polymeric structures (Figure 4A).

Here it is interesting to note that also the isoforms still containing the TMD seem to produce the same kind of intracellular filament structures. Schaeffer et al. could not show colocalization of the intracellular filaments of Uromodulin with the ER marker Calnexin [23]. For ZPLD1 mutants we further checked if an overlapping staining of ZPLD1 with an anti-GM130 antibody, a Golgi marker, is detectable. This was not the case, and we therefore conclude that the observed intracellular ZPLD1 complexes are no longer part of the vesicular transport system (Figure 4B).

### 2.4. Release of the C-Terminal Pro-Peptide and Hence Loss of the Putative EHP Is a Prerequisite for Proper Extracellular Polymerization

**Figure 5:**
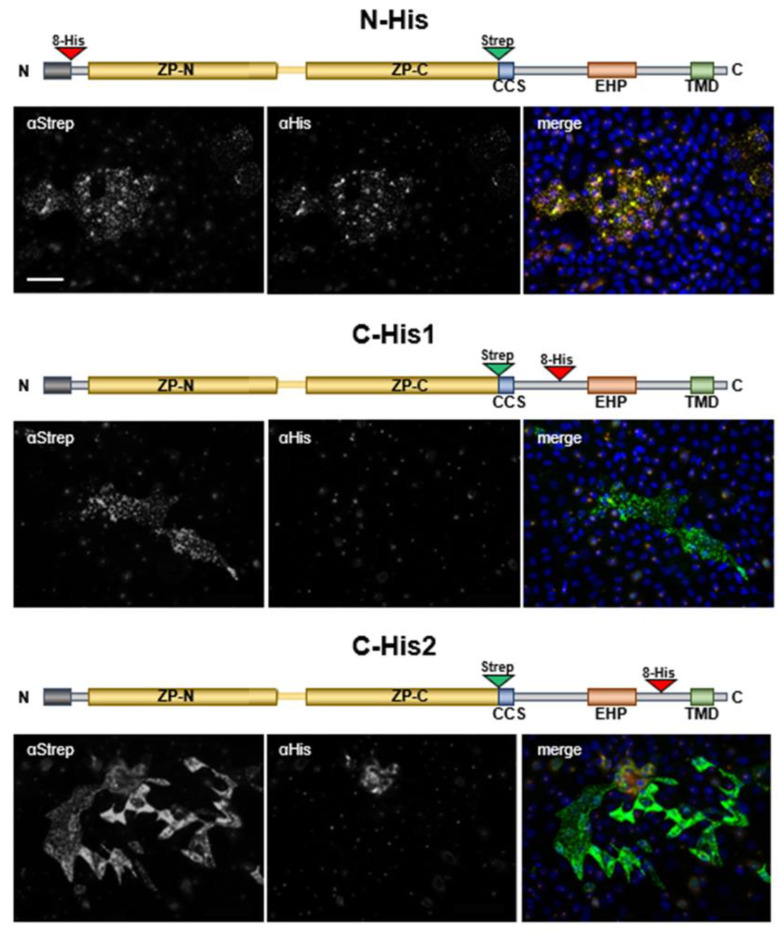
The EHP motif is lost upon ZPLD1 maturation and assembly into filaments. Immunofluorescence analysis on MDCK cells stably expressing N- and C-terminally tagged ZPLD1. A schematic representation of each tagged isoform is shown above the respective immunofluorescence panel. ZPLD1 structure is depicted as before. The EHP motif is indicated as an orange box, the position of 8xHis and Strep-tags as red and green triangles, respectively. Immunofluorescence analysis was carried out on unpermeabilized PFA-fixed cells that were stained for the His-tag and the Strep-tag simultaneously. In the merged picture, the Strep-tag signal is shown in green whereas the 8xHis-tag signal is shown in red. ZPLD1 polymers are positive for both antibodies when the 8xHis-tag is present at the N terminus (N-His). However, the His epitope not detectable when located downstream of the CCS, either before (C-His1) or after (C-His2) the EHP motif. These data suggest that the EHP sequence is lost in polymeric ZPLD1 proteins. Bar, 50 µm.

As already suggested for other ZP proteins and here for ZPLD1 it seems likely that the two hydrophobic motifs are functionally related and necessary to keep the ZP module in an inactive conformation that prevents intracellular polymerization. Next, we assessed whether this regulatory function is lost by cleavage at the CCS that releases the EHP motif. To address this question, we generated different insertions to the wild-type ZPLD1 sequence generating a N-terminal His-tag (N-His), a C-terminal His-tag upstream of the EHP motif (between S_325_ and G_326_) (C-His1), and another variant carrying a C-terminal His-tag downstream of the EHP motif (between S_348_ and N_349_) (C-His2) (Figure 5). These isoforms were as well stably expressed in MDCK cells.

Immunofluorescence experiments with nonpermeabilized cells showed the presence of polymers at the plasma membrane of all constructs when using an antibody against the Strep-tag positioned immediately upstream of the CCS. These polymers looked identical to the ones observed in stable clones expressing untagged wild type ZPLD1, suggesting that the presence of N- and C-terminal His-tags do not interfere with protein processing and assembly into filaments. Only polymers containing the N-His isoform were positive for an anti-His antibody, suggesting that sequence downstream of S_325_, i.e., including the EHP motif, is lost in polymeric ZPLD1.

These findings support the hypothesis, that ZPLD1s’s EHP and IHP motifs have the same function as described for other ZP proteins and that loss of the EHP motif is essential for ZPLD1 as for other ZP-proteins to enable polymerization into extracellular filaments.

## 3. Discussion

ZPLD1 was identified as a main component of the cupula in the inner ear and belongs to the family of the ZP proteins. Most of these members share the ability to polymerize extracellularly into filaments and matrices via their ZP module, while distinct additional functionalities can be attributed [14]. In this work, we aimed at investigating if ZPLD1 is able to form homopolymers and gain new insights into the molecular mechanism that regulates this protein processing. Previous studies performed on murine ZP3 protein and recombinant Uromodulin partly served as a blueprint for our experiments regarding ZPLD1 stepwise maturation [18,23,24]. A combination of genetic engineering, protein expression in a suitable cell line and immunofluorescence microscopy was necessary to identify and initially describe the function of two hydrophobic patches involved in the regulation of ZPLD1 module-mediated interaction and polymerization. The location of these patches, an IHP as part of the bi-partite ZP module and an EHP located distinct at the C-terminus is highly conserved between ZP proteins in terms of secondary structure as well as amino acid properties [23]. Based on these characteristics we identified EHP and IHP motifs in the zebrafish ZPLD1 sequence. To the best of our knowledge, this is the first time that ZPLD1 function and ability to polymerize is described. This greatly supports the hypothesis that this protein is a major functional component of the cupula hydrogel with great importance for proper vestibular function.

To understand if the regulated polymerization follows a universal principle, we analyzed phenotypic characteristics induced by mutations deleting the EHP and IHP of ZPLD1 using the established cellular model of MDCK cells. The observed results for ZPLD1 variants lacking one of the hydrophobic areas mirrors almost exactly prior results of Uromodulin variants [23]. The presence of both hydrophobic patches is required for proper polymerization, which strongly supports the hypothesis of their regulatory function. Already for Uromodulin the interesting additional examination has been reported that variants lacking the EHP traffic to the plasma membrane but seem to stick there without further processing. Interaction of the hydrophobic areas seems to be a prerequisite for the following proteolytic cleavage. A special conformational interaction between the two hydrophobic patches could expose the recognition site (CCS) of ZPLD1 for a so far unidentified protease or an environment that would activate cleavage [23]. Unfortunately, we were not yet able to confidently investigate conditioned medium and cell lysate via Western blot analysis to further justify the lack of secretion of the variant depleted of its putative EHP. We therefore tested this hypothesis by insertion and detection of additional His-tags shortly before and after the identified putative EHP regions. The results indicate that, whilst the EHP seems to be important to enable proteolytic cleavage, it is lost upon ZPLD1 assembly. The EHP release allows for protein polymerization into filaments. This step seems to be a universally valid as it even occurs in soluble ZP module proteins. Prominent examples are some fish vitelline envelope proteins. Secreted as precursors by liver cells the pro-proteins undergo proteolytic cleavage including loss of the EHP upon their arrival at the egg where they subsequently assemble into the egg coat [25,26].

These observations would furthermore seem to support the hypothesis that polymer formation is mediated mainly by the interaction of the internal hydrophobic patches of the ZP modules. Contrastingly, immunofluorescence pictures of permeabilized cells transfected with mutant ZPLD1 variants show internal filament formation even for ZPLD1 lacking its IHP. Intracellular polymerization of variants lacking the IHP were already shown for Uromodulin, interestingly however, Uromodulin lacking its EHP did not form intracellular polymers unless it was further depleted for its membrane anchoring domain [23]. Our findings for ZPLD1 showed otherwise internal polymerization for the variant lacking its IHP independently of the availability of its membrane anchoring domain. Furthermore, observed intracellular polymeric structures were more complex, widely extended over the whole cell and could not be co-located with the Golgi marker GM130. For Uromodulin the ER and Golgi markers calnexin and giantin do not colocalize with intracellular polymers thereby suggesting that the intracellular filaments are likely formed either in the intracellular compartment or in the cytoplasm after retro translocation from the ER [23]. Our findings and the sheer size of the filaments rather support the latter hypothesis and point to a vesicular escape of ZPLD1variants lacking one of its hydrophobic regulating patches.

In summary, our work investigated for the first-time polymerization and underlying mechanisms in ZPLD1. We demonstrated that short hydrophobic motifs common for ZP proteins could also be detected in ZPLD1 using proposed conserved amino acid features. These regions play a similar role for regulation and polymerization as for the classical ZP module proteins. Based on these findings we hypothesize a model for ZPLD1 regulation and processing that is heavily reliant on the interplay of the hydrophobic motifs. Interaction between the patches results in a conformational state, which obviously enables proper attachment to the vesicular membrane during transport to the cytoplasmic membrane (CM). Anchoring of immature ZPLD1 to the CM is a prerequisite for further maturation. Cleavage of the C-terminal pro-peptide, which includes the EHP, by a so-far-unknown protease, results in the release of mature ZPLD1 and paves the way for extracellular protein polymerization. Interplay of the hydrophobic patches avoids intracellular polymerization and shields the protein for intermolecular self-assembly.

This hypothesis is further supported by structural findings for other ZP proteins based on X-ray and Cryo-EM studies. Results indicate that conformational changes and domain swapping events likely play a major role in ZP protein maturation and polymerization [20,21,22]. The EHP of ZP3 has for example been shown to block premature protein polymerization by acting as a “molecular glue” that keeps the ZP module in a conformation essential for secretion while hindering the formation of higher-order structures [22]. Assessment of AlphaFold structure prediction of ZPLD1 however, does not suggest an intramolecular interaction of its hydrophobic patches (Supplementary Figure S3). While IHP and EHP do not seem to interact within a single ZPLD1 molecule their exposed location and orientation could instead enable homodimerization. Studies on crystal structures of Uromodulin already revealed homodimerization mediated by its hydrophobic patches as prerequisite for secretion, processing and subsequent filament formation [21].

Another possible explanation for the lacking proximity of the identified hydrophobic patches in the structural model becomes evident when comparing the interdomain linker sequences of ZPLD1 and Uromodulin (Supplementary Figure S4). Bokhove and colleagues described a rigid interdomain linker consisting of an alpha helix (α1), a beta strand (β1) and the consecutive IHP responsible for maintaining Uromodulin in a polymerization-competent conformation [21]. Sequence alignment reveals that Schaeffer´s proposed model for the prediction of the IHP as well as the relevant hydrophobic sequence we identified for ZPLD1 might correspond to the α1 rather than the internal hydrophobic patch. Consensus of the linker structure seems furthermore likely as linker arrangements have been shown to coincide with the different polymerization abilities of the corresponding ZP proteins [21]. Uromodulin, glycoprotein 2 and α-tectorin sharing rigid, highly structured linkers are all able to homopolymerize as shown for ZPLD1 in this study as well [21]. However, the secondary structure predictions are inconsistent. AlphaFold as, well as four out of five algorithms used for the structural predictions to identify the putative IHP, do not anticipate a helix for the identified hydrophobic sequence region (Figure 2). This fact underlines uncertainties posed by the sole availability of computational structure predictions. Further investigation will be needed to provide more evidence and details to the structural arrangement of the interdomain linker of ZPLD1 and its role in the regulated polymerization process, which is likely common for many, if not all of ZP-proteins. ZPLD1 is an interesting target for further investigations as it seems not solely of great relevance for proper vestibular function but in addition to further adhesive biological functions.

## 4. Materials and Methods

### 4.1. Protein Sequence Analyses

Sequence homologies as well as visual multiple sequence alignments were calculated and created using CLUSTALW (https://www.genome.jp/tools-bin/clustalw last accessed on 10 November 2022) [27]. Secondary structure prediction was carried out using PSIPred (http://bioinf.cs.ucl.ac.uk/psipred/ last accessed on 10 November 2022) [28], Jpred4 (https://www.compbio.dundee.ac.uk/jpred/ last accessed on 10 November 2022) [29], Porter 5.0 (http://distilldeep.ucd.ie/porter/ last accessed on 10 November 2022) [30], Sspro (http://download.igb.uci.edu/sspro4.html last accessed on 10 November 2022) [31], and PredictProtein (https://predictprotein.org/ last accessed on 10 November 2022) [32]. Structure of zebrafish ZPLD1 was predicted by AlphaFold (AlphaFold DB version 2022–06–01) and visualized using pyMOL (The PyMOL Molecular Graphics System, Version 2.5.4 Schrödinger, LLC.) [33,34,35].

### 4.2. ZPLD1 Expression Constructs

Wild-type zebrafish ZPLD1 DNA cloned into vector pEGFP-N1 was purchased from General Biosystems (Durham, NC, USA). The full sequence can be found in the Supplementary Information (Supplementary Figure S5). All genetic manipulations (deletion and insertions) were performed using the Q5 Site-Directed Mutagenesis Kit (New England Biolabs, Ipswich, MA, USA) in combination with a back-to-back primer design. The back-to-back primer design allowed for deletions generated simply by positioning both 5′ ends of forward and reverse primers directly on the sequence flanking the desired deletion. For insertions, additional DNA was added to the 5′ ends of forward and/or reverse primers. A linear double strand was generated while the vector was used as a template. In a subsequent reaction phosphorylation at 5′ ends and vector ligation were performed. Addition of enzyme *Dpn*I cutted methylated template DNA and therefore enhanced transformation efficiency. Primers were ordered from Metabion international AG (Planegg/Steinkirchen, Germany). Primer sequences can be found in Supplementary Table S1. The Strep-tag was inserted between amino acids K315 and R316 with a short SSGS linker anterior to the Strep sequence. The putative EHP was deleted between amino acids V333 and R342 The putative IHP was deleted between amino acids E_360_ and T_366_. The TMD domain was deleted between amino acids V366 and S_390_. The 8xHis-tag was inserted between amino acids Q20 and F21 with a short SSGS linker posterior to the 8xHis-tag (N-His), between amino acids S_325_ and G_326_ (C-His1), and in-between S_348_ and N_349_ (C-His2) respectively. All constructs were sequence-verified before transfection.

### 4.3. Cell Lines and Culture Conditions

Madin–Darby canine kidney (MDCK NBL-2) cells were purchased from ATCC (Manassas, VA, USA) and grown in DMEM supplemented with 10% fetal bovine serum, 100 U/mL penicillin, 100 U/mL streptomycin, 25 mM D-glucose at 37 °C, 5% CO_2_. Stable clones were generated by transfecting MDCK cells by using Lipofectamine 3000 (Invitrogen, Thermo Fisher Scientific, Waltham, MA, USA) following the manufacturer’s protocol. Selection was started 24 h after transfection by adding 1 mg/mL Geniticin (G418 sulfate) (Invitrogen, Thermo Fisher Scientific, Waltham, MA, USA) and was pursued for 1–3 weeks to obtain a G418 resistant cell population.

### 4.4. Protein Purification and Western Blot

MDCK cells stably expressing Strep-tagged ZPLD1 were grown on 100 mm polystyrene cell culture dishes (Corning, Corning, NY, USA). As soon as cells reached confluence the medium was collected and stored at −20 °C until further use for purification.

A total of 1 mL of Strep-tactin slurry (Iba Lifesciences, Göttingen, Germany) was transferred to a reusable gravity flow column. Collected medium was thawed, centrifuged for 20 min at 1400× *g* to spin down any contained cells and cell components and pH measured using a pH test strip (Merck KGaA, Darmstadt, Germany). Pooled media with a pH between 7 and 8 was run over the column and purification conducted according to the manufacturer´s protocol.

Elution fractions were pooled, concentrated using an Amicon Ultra 0.5 mL 10 K centrifugal filter device (Merck KGaA, Darmstadt, Germany), measured at A280 (NanoDrop, Thermo Fisher Scientific, Waltham, MA, USA) and stored at 4 °C.

Purified ZPLD1 was deglycosylated under denaturing conditions using PNGase *F* (New England Biolabs, Ipswich, MA, USA) according to the manufacturer´s protocol. Deglycosylated protein was reduced in Laemmli buffer containing 2.5% or 5% mercaptoethanol at 96 °C for 5 or 15 min and applied to a 12% SDS-polyacrylamide gel electrophoresis (PAGE). After blotting the nitrocellulose membrane (GE Healthcare, General Electric, Boston, MA, USA) was incubated (1:10,000) with polyclonal guinea pig antibodies against two distinct ZPLD1 peptide sequences, as previously described [1]. A secondary horseradish peroxidase-conjugated antibody (1:4000 dilution, Jackson ImmunoResearch Inc., West Grove, PA, USA) was subsequently applied for visualization.

An uncropped image of the Western blot detecting PNGase *F* treated ZPLD1 purified from cell culture media of transfected MDCK cells is shown in Supplementary Figure S6. A Western blot detecting untreated ZPLD1 as well as a negative control mock transfected sample can be found in Supplementary Figure S6 as well.

### 4.5. Immunofluorescence

Cells grown on 8 well Lab-Tek II chamber slides (Thermo Fisher Scientific, Waltham, MA, USA) were fixed in 4% paraformaldehyde (PFA) for 30 min. When needed, cells were permeabilized 30 min at room temperature with 0.5% Triton X-100. After washing in PBS, cells were incubated 30 min at room temperature in 10% goat serum in PBS. Cells were then incubated for 2 h at room temperature with the mouse anti-Strep-tag II monoclonal antibody (1:100 dilution; Iba Lifesciences, Göttingen, Germany) and where needed simultaneously with the rabbit anti-His-tag antibody (1:400 dilution; Invitrogen, Thermo Fisher Scientific, Waltham, MA, USA). Permeabilized cells were simultaneously co-stained with rabbit anti-GM130 Golgi family protein antibody (1:100 dilution; Abcam, Cambridge, UK). Cells were washed in PBS and incubated for 1 h at room temperature with the appropriate secondary antibody: Alexa-Fluor 594-conjugated goat secondary antibody against rabbit immunoglobulin G (IgG) (dilution 1:400; Abcam, Cambridge, UK); or Alexa-Fluor 488-conjugated goat antibody against mouse IgG (1:200 dilution; Abcam, Cambridge, UK). Cells were then stained for 5 min with 4,6-diamidino-2-phenylindole (DAPI) and mounted using Shandon Immu-Mount (Thermo Fisher Scientific, Waltham, MA, USA). All slides were visualized under a Zeiss AxioCam MRm fluorescence microscope. Images were evaluated with the AxioVision 4.8.2 SP3 program (Carl Zeiss AG, Oberkochen, Germany).

Control immunofluorescence images of untransfected MDCK cells can be found in the Supplementary Materials (Supplementary Figure S7).

## Data Availability

Not applicable.

## Supplementary Materials

The following supporting information can be downloaded at: https://www.mdpi.com/article/10.3390/ijms232213894/s1, Figure S1: Schematic representation of the localization of the cupula in the (human) inner ear [1]; Figure S2: Visual multiple sequence alignment using CLUSTAL W; Figure S3: Visualization of predicted structure of zebrafish ZPLD1; Figure S4. Sequence comparison of the interdomain linker sequences of zebrafish ZPLD1 and human Uromodulin; Figure S5: Full sequence of initially ordered expression vector; Figure S6: Western blot detecting PNGase *F* treated as well as untreated ZPLD1; Figure S7: Control immunofluorescence images of untransfected MDCK cells; Table S1: Primers (5′-3′) used to generate ZPLD1 expression constructs.
