# Supplementary material for: Zona Pellucida like Domain Protein 1 (ZPLD1) Polymerization Is Regulated by Two Distinguished Hydrophobic Motifs
Source: Int J Mol Sci. 2022 Nov 11;23(22):13894. doi: 10.3390/ijms232213894 (PMC9692296; doi:10.3390/ijms232213894)
Supplement: Supplementary file 1 [file ijms-23-13894-s001.zip › ijms-1964402-supplementary.pdf]

International Journal of
Molecular Sciences

MDPI

Supplementary Materials:

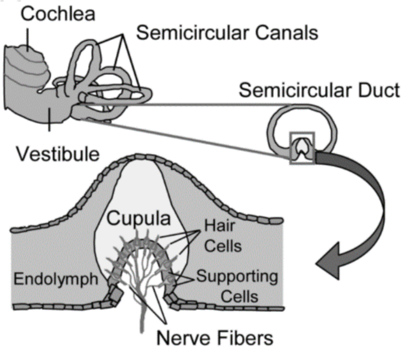

Supplementary Figure S1. Schematic representation of the localization of the cupula in the (human) inner ear [1]. Figure reused with permission from Ref. [1].

```
CLUSTAL 2.1 multiple sequence alignment


Zebrafish       MERLCVILLLVSKTFIANAQFNGFNCDANFHSRFPAERDISVYCGVQTITLKINFCPVLF 60
Salmon          MEQICLIILLISKALSVGAQFNGYNCDANFHSRFPAERDISVYCGVQTITLKINFCPVLF
Human           MEQIWLLLLLTIRVLPGSAQFNGYNCDANLHSRFPAERDISVYCGVQAITMKINFCTVLF
                **:: :::**  :.:  .**:*****:*****************:**:*****.***

Zebrafish       SGYTDTDLALNGRHGDAHCRGFINNNTFPTVVLFSISLNTLESCGNSLVVSTAHGPNAYG 120
Salmon          SGYTDTDLALNGRHGDAHCRGFINNNTFPTVVLFSISLATLETCGNALVVSTAQGPNAYG
Human           SGYSETDLALNGRHGDSHCRGFINNNTFPAVVIFIINLSTLEGCGNNLVVSTIPGVSAYG
                ***::*****:*****:***********:**:* *.* *** *** ***  * .***

Zebrafish       NLSLVQIGNIAGYIDTPDPPTVISYLPGLLYKFSCSYPLEYLVNNTQLASSAAAISVKDS 180
Salmon          NLSLVQIGNISGYIDTPDPPTIISYLPGLLYKFSCSYPLEYLVNNTQLASSAAAISVKDS
Human           NATSVQVGNISGYIDTPDPPTIISYLPGLLYKFSCSYPLEYLVNNTQLASSSAAISVREN
                * : **:***:**********:*****************************:*****::.

Zebrafish       NGTFVSTLNLLLYNDSSYIQNLAIPMSGLTLKTRVFAAVKATNLDRRWNVLMDYCYTTPS 240
Salmon          NGTFVSTLSLLLYNDSSYVQQLSIPMAGLTLKTRVFAAVKATNLDRRWNVLMDYCYTTAS
Human           NGTFVSTLNLLLYNDSTYNQQLIIPSIGLPLKTKVFAAVQATNLDGRWNVLMDYCYTTPS
                ********.*******:* *:* **  **.***:*****:***** ************.*

Zebrafish       GNPNDELRYDLFFGCDKDPQTTVFENGKSQMGRFSFEVFRFVKHKNQKMSTVFLHCVTKL 300
Salmon          GNPNDELRYDLFFSCDKDPQTTVFENGKSQMGRFAFEVFRFVKHKNQKMSTVFLHCVTKL
Human           GNPNDDIRYDLFLSCDKDPQTTVIENGRSQRGRFSFEVFRFVKHKNQKMSTVFLHCVTKL
                *****::*****:.*********:***:** ***:*************************

Zebrafish       CRSDDCPMLLPICGKRKKRDVSERS----GGASDNAVITAGPIITRSDETPSNISQLAQL 356
Salmon          CRADDCPMLMPICGSRKKRDVSERTES--NSASGNAIITAGPIITRSDDTPMNNSQLAQL
Human           CRADDCPFLMPICSHRERRDAGRRTTWSPQSSSGSAVLSAGPIITRSDETPTNNSQLGSP
                **:****:*:***. *::**...*:     .:*..*:::********:** * *** ..

Zebrafish       NGPPFKMNSVTSALISAIAILAVMSMCFFILSMSLLRGKQTPATSLSGSHNPAFS 411
Salmon          NSPVFRMNTVTSALISGIIILGVMSLCFFILSLTLLKGKRAPPTILSGARNPAFN
Human           SMPPFQLNAITSALISGMVILGVTSFSLLLCSLALLHRKGPTSLVLNGIRNPVFD
                . * *::*::******.: **.* *:.::: *::**: * ...  *.* :**.*.
```

Supplementary Figure S2. Visual multiple sequence alignment using CLUSTAL W (https://www.genome.jp/tools-bin/clustalw) [27]. Zebrafish (accession A0A0R4ITH5), salmon (accession ACN10635, ~86.4% identity) and human (accession AAH31261, ~71.5% identity) ZPLD1 amino acid sequences are highly conserved.

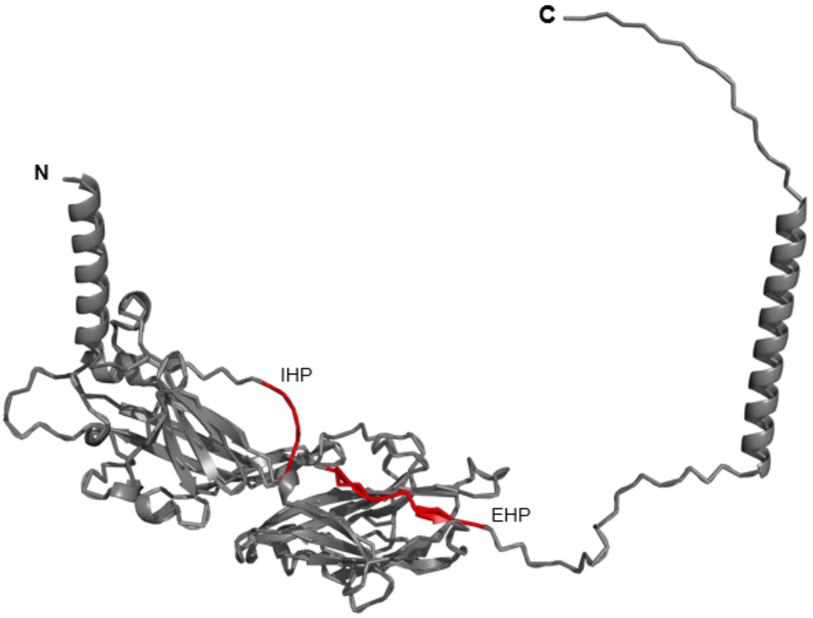

Supplementary Figure S3. Visualization of predicted structure of zebrafish ZPLD1 (accession A0A0R4ITH5). Model prediction by AlphaFold (AlphaFold DB version 2022-06-01) [33,34]. Putative IHP and EHP are marked in red.

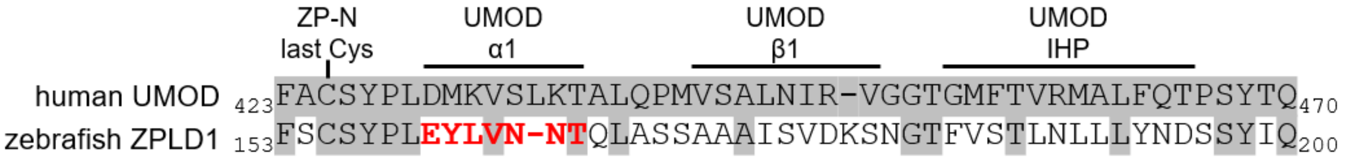

Supplementary Figure S4. Sequence comparison of the interdomain linker sequences of zebrafish ZPLD1 (accession A0A0R4ITH5) and human Uromodulin (accession P07911). The structured rigid linker region of Uromodulin consists of an α-helix (α1), a beta-strand (β1) and the consecutive IHP [21]. Alignment reveals that Schaeffer´s proposed model for the prediction of the IHP as well as the relevant hydrophobic sequence we identified for ZPLD1 (marked in red) might correspond to the α1-helix rather than the internal hydrophobic patch.

TAGTTATTAATAGTAATCAATTACGGGGTCATTAGTTCATAGCCCATATATGGAGTTCCGCGTTACATAACTTACGGTAAATGGCCCGCCTGGCTGACCGCCCAACGAC

CCCCGCCCATTGACGTCAATAATGACGTATGTTCCCATAGTAACGCCAATAGGGACTTTCCATTGACGTCAATGGGTGGAGTATTTACGGTAAACTGCCCACTTGGCAG

TACATCAAGTGTATCATATGCCAAGTACGCCCCCTATTGACGTCAATGACGGTAAATGGCCCGCCTGGCATTATGCCCAGTACATGACCTTATGGGACTTTCCTACTTG

GCAGTACATCTACGTATTAGTCATCGCTATTACCATGGTGATGCGGTTTTGGCAGTACATCAATGGGCGTGGATAGCGGTTTGACTCACGGGGATTTCCAAGTCTCCAC

CCCATTGACGTCAATGGGAGTTTGTTTTGGCACCAAAATCAACGGGACTTTCCAAAATGTCGTAACAACTCCGCCCCATTGACGCAAATGGGCGGTAGGCGTGTACGGT

GGGAGGTCTATATAAGCAGAGCTGGTTTAGTGAACCGTCAGATCCGCTAGCCACC**ATGGAACGTTTGTGTGTGATTCTCTTGCTTGTAAGTAAAACTTTCATAGCGAAT**

GCTCAATTCAATGGGTTCAACTGTGATGCCAACTTCCACAGCCGCTTCCCCGCCGAGCGGGACATCAGTGTGTATTGTGGAGTTCAAACCATAACTCTGAAGATTAATT

TTTGCCCGGTGCTTTTTTCTGGCTACACTGACACTGACCTGGCACTGAATGGGCGTCACGGGGATGCCCACTGCAGAGGCTTCATCAACAACAACACGTTCCCCACGGT

TGTGCTGTTTAGCATCAGTCTCAACACTCTGGAGTCTTGCGGAAACTCACTGGTGGTCTCTACGGCTCATGGGCCCAACGCCTATGGGAATCTTTCCCTGGTCCAGATT

GGGAATATAGCAGGATACATTGACACTCCGGATCCCCCGACTGTCATCAGTTACCTACCAGGACTGCTTTACAAGTTCAGTTGTAGCTACCCACTGGAATACCTGGTCA

ACAACACACAGCTTGCTTCGTCGGCCGCAGCGATATCAGTGAAGGACAGTAATGGTACTTTTGTTAGCACGCTCAATTTGCTTCTATACAACGACTCCTCATACATTCA

GAACCTCGCCATCCCAATGTCTGGACTTACTCTGAAGACGCGAGTGTTCGCCGCTGTAAAAGCCACCAACCTAGATAGGAGGTGGAACGTTCTGATGGACTACTGTTAC

**ACCACTCCCTCTGGAAATCCTAATGATGAGCTGCGATATGATCTTTTCTTTGGATGCGATAAGGACCCACAGACGACAGTGTTTGAGAACGGGAAGAGCCAGATGGGTC
GCTTCTCCTTTGAGGTGTTTCGCTTTGTCAAGCACAAAAACCAGAAAATGTCGACTGTTTTTCTGCACTGCGTGACCAAGCTGTGTCGCTCTGATGACTGCCCCATGCT
CCTGCCCATCTGCGGTAAGAGGAAGAAAAGGGATGTGTCAGAAAGAAGCGGTGGTGCCTCAGACAATGCAGTTATAACCGCTGGACCCATCATCACCCGAAGTGATGAA
ACTCCCAGTAATATATCCCAACTAGCGCAGTTAAACGGCCCTCCATTCAAAATGAATTCAGTGACGAGCGCTTTAATTTCTGCCATCGCGATCCTCGCCGTTATGAGCA
TGTGTTTCTTCATCTTGTCTATGTCGCTGCTGAGGGGAAAACAGACGCCAGCAACATCTCTATCAGGATCCCACAACCCAGCGTTCAGC**CTCGAGCTCAAGCTTCGAAT
TCTGCAGTCGACGGTACCGCGGGCCCGGGATCCACCGGTCGCCACCATGGTGAGCAAGGGCGAGGAGCTGTTCACCGGGGTGGTGCCCATCCTGGTCGAGCTGGACGGC
GACGTAAACGGCCACAAGTTCAGCGTGTCCGGCGAGGGCGAGGGCGATGCCACCTACGGCAAGCTGACCCTGAAGTTCATCTGCACCACCGGCAAGCTGCCCGTGCCCT
GGCCCACCCTCGTGACCACCCTGACCTACGGCGTGCAGTGCTTCAGCCGCTACCCCGACCACATGAAGCAGCACGACTTCTTCAAGTCCGCCATGCCCGAAGGCTACGT
CCAGGAGCGCACCATCTTCTTCAAGGACGACGGCAACTACAAGACCCGCGCCGAGGTGAAGTTCGAGGGCGACACCCTGGTGAACCGCATCGAGCTGAAGGGCATCGAC
TTCAAGGAGGACGGCAACATCCTGGGGCACAAGCTGGAGTACAACTACAACAGCCACAACGTCTATATCATGGCCGACAAGCAGAAGAACGGCATCAAGGTGAACTTCA
AGATCCGCCACAACATCGAGGACGGCAGCGTGCAGCTCGCCGACCACTACCAGCAGAACACCCCCATCGGCGACGGCCCCGTGCTGCTGCCCGACAACCACTACCTGAG
CACCCAGTCCGCCCTGAGCAAAGACCCCAACGAGAAGCGCGATCACATGGTCCTGCTGGAGTTCGTGACCGCCGCCGGGATCACTCTCGGCATGGACGAGCTGTACAAG
TCCGGACTCAGATCTATGGAGACCCCGTCCCAGCGGCGCGCCACCCGCAGCGGGGCGCAGGCCAGCTCCACTCCGCTGTCGCCCACCCGCATCACCCGGCTGCAGGAGA
AGGAGGAGGATCCACCGGATCTAGATAACTGATCATAATCAGCCATACCACATTTGTAGAGGTTTTACTTGCTTTAAAAAACCTCCCACACCTCCCCCTGAACCTGAAA
CATAAAATGAATGCAATTGTTGTTGTTAACTTGTTTATTGCAGCTTATAATGGTTACAAATAAAGCAATAGCATCACAAATTTCACAAATAAAGCATTTTTTTCACTGC
ATTCTAGTTGTGGTTTGTCCAAACTCATCAATGTATCTTAACGCGTAAATTGTAAGCGTTAATATTTTGTTAAAATTCGCGTTAAATTTTTGTTAAATCAGCTCATTTT
TTAACCAATAGGCCGAAATCGGCAAAATCCCTTATAAATCAAAAGAATAGACCGAGATAGGGTTGAGTGTTGTTCCAGTTTGGAACAAGAGTCCACTATTAAAGAACGT
GGACTCCAACGTCAAAGGGCGAAAAACCGTCTATCAGGGCGATGGCCCACTACGTGAACCATCACCCTAATCAAGTTTTTTGGGGTCGAGGTGCCGTAAAGCACTAAAT
CGGAACCCTAAAGGGAGCCCCCGATTTAGAGCTTGACGGGGAAAGCCGGCGAACGTGGCGAGAAAGGAAGGGAAGAAAGCGAAAGGAGCGGGCGCTAGGGCGCTGGCAA
GTGTAGCGGTCACGCTGCGCGTAACCACCACACCCGCCGCGCTTAATGCGCCGCTACAGGGCGCGTCAGGTGGCACTTTTCGGGGAAATGTGCGCGGAACCCCTATTTG
TTTATTTTTCTAAATACATTCAAATATGTATCCGCTCATGAGACAATAACCCTGATAAATGCTTCAATAATATTGAAAAAGGAAGAGTCCTGAGGCGGAAAGAACCAGC
TGTGGAATGTGTGTCAGTTAGGGTGTGGAAAGTCCCCAGGCTCCCCAGCAGGCAGAAGTATGCAAAGCATGCATCTCAATTAGTCAGCAACCAGGTGTGGAAAGTCCCC
AGGCTCCCCAGCAGGCAGAAGTATGCAAAGCATGCATCTCAATTAGTCAGCAACCATAGTCCCGCCCCTAACTCCGCCCATCCCGCCCCTAACTCCGCCCAGTTCCGCC
CATTCTCCGCCCCATGGCTGACTAATTTTTTTTATTTATGCAGAGGCCGAGGCCGCCTCGGCCTCTGAGCTATTCCAGAAGTAGTGAGGAGGCTTTTTTGGAGGCCTAG
GCTTTTGCAAAGATCGATCAAGAGACAGGATGAGGATCGTTTCGCATGATTGAACAAGATGGATTGCACGCAGGTTCTCCGGCCGCTTGGGTGGAGAGGCTATTCGGCT
ATGACTGGGCACAACAGACAATCGGCTGCTCTGATGCCGCCGTGTTCCGGCTGTCAGCGCAGGGGCGCCCGGTTCTTTTTGTCAAGACCGACCTGTCCGGTGCCCTGAA
TGAACTGCAAGACGAGGCAGCGCGGCTATCGTGGCTGGCCACGACGGGCGTTCCTTGCGCAGCTGTGCTCGACGTTGTCACTGAAGCGGGAAGGGACTGGCTGCTATTG
GGCGAAGTGCCGGGGCAGGATCTCCTGTCATCTCACCTTGCTCCTGCCGAGAAAGTATCCATCATGGCTGATGCAATGCGGCGGCTGCATACGCTTGATCCGGCTACCT
GCCCATTCGACCACCAAGCGAAACATCGCATCGAGCGAGCACGTACTCGGATGGAAGCCGGTCTTGTCGATCAGGATGATCTGGACGAAGAGCATCAGGGGCTCGCGCC
AGCCGAACTGTTCGCCAGGCTCAAGGCGAGCATGCCCGACGGCGAGGATCTCGTCGTGACCCATGGCGATGCCTGCTTGCCGAATATCATGGTGGAAAATGGCCGCTTT
TCTGGATTCATCGACTGTGGCCGGCTGGGTGTGGCGGACCGCTATCAGGACATAGCGTTGGCTACCCGTGATATTGCTGAAGAGCTTGGCGGCGAATGGGCTGACCGCT
TCCTCGTGCTTTACGGTATCGCCGCTCCCGATTCGCAGCGCATCGCCTTCTATCGCCTTCTTGACGAGTTCTTCTGAGCGGGACTCTGGGGTTCGAAATGACCGACCAA
GCGACGCCCAACCTGCCATCACGAGATTTCGATTCCACCGCCGCCTTCTATGAAAGGTTGGGCTTCGGAATCGTTTTCCGGGACGCCGGCTGGATGATCCTCCAGCGCG
GGGATCTCATGCTGGAGTTCTTCGCCCACCCTAGGGGGAGGCTAACTGAAACACGGAAGGAGACAATACCGGAAGGAACCCGCGCTATGACGGCAATAAAAAGACAGAA
TAAAACGCACGGTGTTGGGTCGTTTGTTCATAAACGCGGGGTTCGGTCCCAGGGCTGGCACTCTGTCGATACCCCACCGAGACCCCATTGGGGCCAATACGCCCGCGTT
TCTTCCTTTTCCCCACCCCACCCCCCAAGTTCGGGTGAAGGCCCAGGGCTCGCAGCCAACGTCGGGGCGGCAGGCCCTGCCATAGCCTCAGGTTACTCATATATACTTT
AGATTGATTTAAAACTTCATTTTTAATTTAAAAGGATCTAGGTGAAGATCCTTTTTGATAATCTCATGACCAAAATCCCTTAACGTGAGTTTTCGTTCCACTGAGCGTC
AGACCCCGTAGAAAAGATCAAAGGATCTTCTTGAGATCCTTTTTTTCTGCGCGTAATCTGCTGCTTGCAAACAAAAAAACCACCGCTACCAGCGGTGGTTTGTTTGCCG
GATCAAGAGCTACCAACTCTTTTTCCGAAGGTAACTGGCTTCAGCAGAGCGCAGATACCAAATACTGTCCTTCTAGTGTAGCCGTAGTTAGGCCACCACTTCAAGAACT
CTGTAGCACCGCCTACATACCTCGCTCTGCTAATCCTGTTACCAGTGGCTGCTGCCAGTGGCGATAAGTCGTGTCTTACCGGGTTGGACTCAAGACGATAGTTACCGGA
TAAGGCGCAGCGGTCGGGCTGAACGGGGGGTTCGTGCACACAGCCCAGCTTGGAGCGAACGACCTACACCGAACTGAGATACCTACAGCGTGAGCTATGAGAAAGCGCC
ACGCTTCCCGAAGGGAGAAAGGCGGACAGGTATCCGGTAAGCGGCAGGGTCGGAACAGGAGAGCGCACGAGGGAGCTTCCAGGGGGAAACGCCTGGTATCTTTATAGTC

CTGTCGGGTTTCGCCACCTCTGACTTGAGCGTCGATTTTTGTGATGCTCGTCAGGGGGGCGGAGCCTATGGAAAAACGCCAGCAACGCGGCCTTTTTACGGTTCCTGGC

CTTTTGCTGGCCTTTTGCTCACATGTTCTTTCCTGCGTTATCCCCTGATTCTGTGGATAACCGTATTACCGCCATGCAT

Supplementary Figure S5. Full sequence of initially ordered expression vector (accession A0A0R4ITH5). The zebrafish ZPLD1 sequence is indicated in bold letters.

Supplementary Table S1. Primers (5′-3′) used to generate ZPLD1 expression constructs

**Mutation**	**Primer**
Deletion of eGFP	Forward: CAGCCATACCACATTTGTAGAGGTTTTACTTGC<br>Reverse: TCACTCGAGGCTGAACGCTGGG
Insertion of the Strepavidin-tag (and corresponding Linker)	Forward: TGGAGCCATCCGCAGTTTGAAAAAAGGAA-GAAAAGGGATGTGTCAGAAAGAAGCGG<br>Reverse: AGAGCCAGAAGACTTACCGCAGATGGGCAGGAGCATGGGGC
Deletion of the putative EHP	Forward: AGTGATGAAACTCCCAGTAATATATCCC<br>Reverse: TGCATTGTCTGAGGCACCACCGC
Deletion of the putative IHP	Forward: CAGCTTGCTTCGTCGGCCGCAGCG<br>Reverse: CAGTGGGTAGCTACAACTGAACTTGTAAAGCAGTCC
Deletion of the putative TMD	Forward: ATGTCGCTGCTGAGGGGAAAACAGACG<br>Reverse: TGAATTCATTTTGAATGGAGGGCCG
Insertion of the N-terminal 8xHis-tag (N-His)	Forward: TCTTCTGGCTCTTTCAATGGGTTCAACTGTGATGCCAACTTCC<br>Reverse: ATGATGATGATGGTGATGGTGATGTTGAGCATTCGC-TATGAAAGTTTTACTTACAAGC
Insertion of the C-terminal 8xHis-tag N-terminal of the EHP (C-His1)	Forward: CATCATCATCATGGTGGTGCCTCAGAC<br>Reverse: GTGATGGTGATGGCTTCTTTCTGACACATCCC
Insertion of the C-terminal 8xHis-tag C-terminal of the EHP (C-His2)	Forward: CATCATCATCATAATATATCCCAACTAGCGCAG<br>Reverse: GTGATGGTGATGACTGGGAGTTTCATCACTTCG

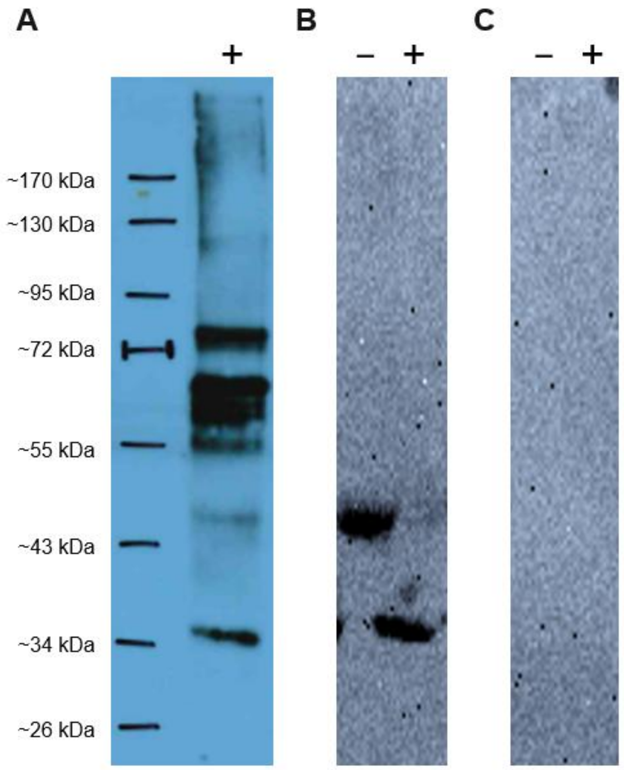

Supplementary Figure S6. Western blot detecting of PNGase F treated (+) as well as untreated (-) ZPLD1. The protein was purified from MDCK cell culture media. (A) Protein reduced and denatured in Laemmli buffer containing 2.5% mercaptoethanol at 96 °C for 5 min Besides the 35 kDa monomer higher molecular weight species can be observed. (B) Protein reduced and denatured in Laemmli buffer containing 5% mercaptoethanol at 96 °C for 15 min to obtain a higher portion of monomeric protein. (C) Control sample obtained from mock transfected MDCK cells.

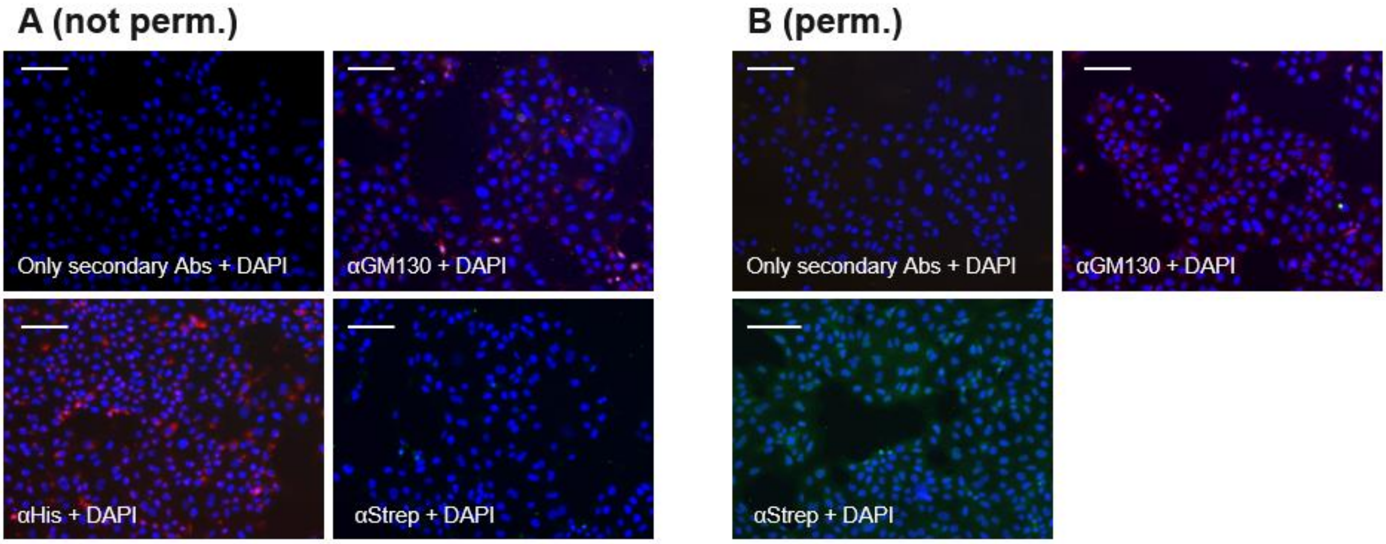

Supplementary Figure S7. Control immunofluorescence images of untransfected MDCK cells. Cells treated with: a mixture of all used secondary antibodies, antibodies against the Golgi marker GM130, and tag antibodies (His/Strep) (see Materials and Methods). Alle samples were additionally stained with DAPI. (A) Cells were not permeabilized; (B) Cells permeabilized using Triton X-100. Bar, 50 µm.
